# Flecainide Toxicity Leading to Transient Heart Failure With Reduced Ejection Fraction: A Case Study

**DOI:** 10.7759/cureus.84100

**Published:** 2025-05-14

**Authors:** Trevor Wolchover, Leili Pourafkari, Vishva Dev, Jasprit Takher

**Affiliations:** 1 Internal Medicine, Los Robles Regional Medical Center, Thousand Oaks, USA; 2 Cardiology, Los Robles Regional Medical Center, Thousand Oaks, USA

**Keywords:** antiarrhythmic drug, cardiology, cardiology imaging, echocardiogram (echo), electrocardiogram (ecg/ekg), flecainide, heart failure and medical education, heart failure with reduced ejection fraction

## Abstract

Flecainide, a class Ic antiarrhythmic, has been widely prescribed for managing cardiac arrhythmias. While effective in rhythm control, there is growing, albeit limited, evidence of its association with reversible heart failure with reduced ejection fraction (HFrEF), particularly under circumstances that may exacerbate drug toxicity. This case report presents a clinically significant episode of flecainide-induced HFrEF in a patient without structural heart disease, supported by a targeted review of the literature. The proposed mechanism involves QRS prolongation and impaired myocardial conduction due to sodium channel blockade, compounded by transient renal dysfunction. Clinicians must remain vigilant for cardiotoxic effects of flecainide, especially in patients with evolving renal function, even in the absence of traditional contraindications.

## Introduction

Flecainide, a class Ic sodium channel blocker, is commonly used to treat supraventricular and ventricular arrhythmias. Its mechanism involves potent blockade of fast inward sodium currents during the depolarization phase of cardiac action potentials, leading to slowed conduction and prolonged refractoriness, particularly in atrial tissue [1,2,3].

Despite its efficacy in rhythm control, the safety of flecainide in certain populations remains a concern [4]. While it is generally contraindicated in patients with structural heart disease or prior myocardial infarction, case reports and observational studies have begun to suggest a rare, but serious association between flecainide and new-onset HFrEF, even in patients without known cardiac dysfunction [5,6,7].

The true incidence of flecainide-induced HFrEF is unknown but appears to be rare. For instance, in a review of 415 patients treated with class Ic antiarrhythmics, only a handful were found to have developed new systolic dysfunction potentially attributable to flecainide [8,9]. Emerging literature suggests that the risk may be higher in the presence of renal impairment, drug interactions, or significant QRS prolongation [6,10,11]. Age, polypharmacy, and female sex may also play a role, though data are limited [12]. This case presents a unique instance of flecainide-induced HFrEF in a patient with normal baseline ejection fraction and renal function who developed acute kidney injury (AKI) and significant QRS widening. The case underscores the importance of early recognition, risk stratification, and renal function monitoring in patients receiving flecainide.

## Case presentation

A 75-year-old female with a past medical history notable for atrial fibrillation, recently started on flecainide and hypertension, presented following a syncopal episode and subsequent fall in the restroom. The patient reported needing assistance to reach the bathroom when she experienced syncope, describing feelings of weakness and lightheadedness.

**Figure 1:**
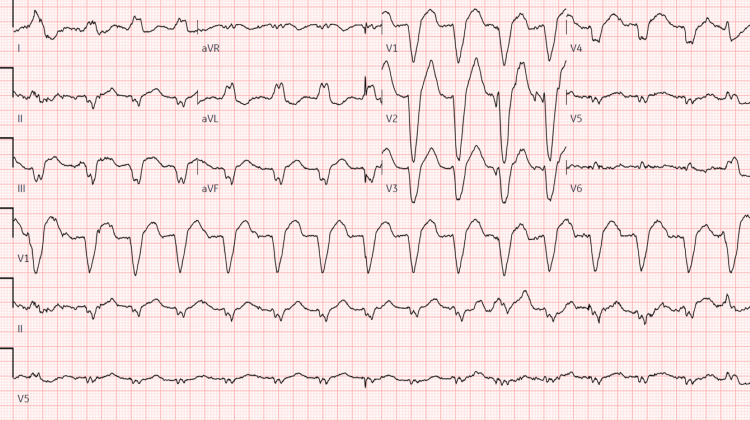
Electrocardiogram on presentation showing wide QRS tachycardia: left axis deviation, left bundle branch block, ventricular rate 102 bpm, QRS duration 192 ms, QT/QTc 494/643 ms

**Figure 2:**
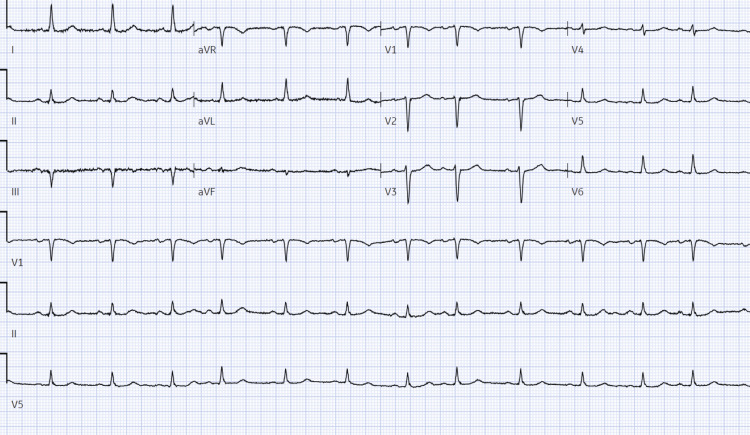
Baseline electrocardiogram prior to initiation of flecainide showing sinus rhythm with premature atrial complexes, otherwise normal ECG, ventricular rate 77 bpm, PR interval 176 ms, QRS duration 92 ms, QT/QTc 418/473 ms

On initial arrival to the emergency department, the patient was afebrile, with a heart rate of 109 bpm, respiratory rate 20 breaths per minute, and blood pressure 139/63 mmHg. The patient’s labs were notable for a white blood cell count of 13.6 10^3^/uL, potassium 3.6 mmol/L, and an acute kidney injury (AKI) with creatinine 1.91 mm/dL, up from her previous baseline of 0.90 mg/dL six months prior. Electrocardiogram (EKG) revealed wide QRS tachycardia with a ventricular rate of 102 bpm and left bundle branch block (LBBB) with a QRS duration of 192 ms (Figure 1). Review of previous medical records indicated an EKG and echocardiogram performed six months prior, demonstrating normal sinus rhythm with a QRS duration of 92 ms and a QTc of 473 (Figure 2), alongside a normal ejection fraction and no structural abnormalities (Video 1), respectively. The patient was admitted for further evaluation, and flecainide was discontinued due to its likely association with increased QRS duration, while Metoprolol succinate was initiated for improved rate control.

**Video 1:** Long-axis parasternal view with normal left ventricle (LV) contractility

**Figure 3:**
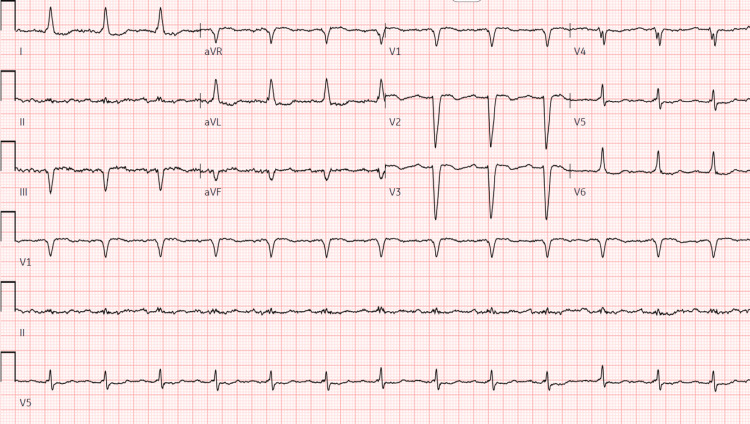
EKG three days after admission and following cessation of flecainide showing sinus rhythm with a first-degree A-V block: non-specific intra-ventricular conduction block, ventricular rate 80 bpm, PR interval 222 ms, QRS duration 130 ms, QT/QTc 428/493 ms

Subsequent echocardiography during admission revealed a decreased ejection fraction of 35-40%, along with a dyskinetic septum and left ventricular hypertrophy (Video 2), prompting cardiac catheterization to rule out coronary artery disease in the setting of a newly reduced ejection fraction. Coronary angiography revealed patent coronary arteries with mild global hypokinesia and moderately reduced left ventricular ejection fraction. Following cessation of flecainide, repeat EKG three days later (Figure 3) demonstrated improvement in QRS duration to 130 ms compared to the previous 192 ms. In addition, repeat echocardiography five days after flecainide cessation exhibited significant improvement in ejection fraction (Video 3).

**Video 2:** Echo on admission: four-chamber view showing left ventricle (LV) dysfunction with left ventricular ejection fraction (LVEF) 35-40% with marked septal dys-synchrony

**Video 3:** Echo five days after discontinuing flecainide with improved left ventricular ejection fraction (LVEF) and dys-synchrony resolved

Upon discharge, the decision was made to discontinue flecainide and maintain treatment with metoprolol succinate, considering flecainide's likely contribution to worsened ejection fraction and prolonged QRS duration.

## Discussion

This case report highlights a clinical scenario of flecainide-induced heart failure with reduced ejection fraction (HFrEF) in a patient with a history of atrial fibrillation. Flecainide, a class Ic antiarrhythmic agent, is commonly used for the management of various cardiac arrhythmias due to its potent sodium channel-blocking properties [1]. Flecainide is known for various cardiotoxic adverse effects, including proarrhythmic effects and, more rarely, negative inotropy leading to heart failure [2,3].

In this case, one significant aspect of the patient's clinical course was the development of AKI, which likely contributed to flecainide accumulation and subsequent toxicity. Flecainide, which has a narrow therapeutic window, is primarily metabolized by the liver, but approximately 30% of the drug is excreted unchanged in the urine [6]. In patients with impaired renal function, the reduced clearance of flecainide can lead to accumulation and increased risk of adverse effects, such as QRS prolongation and ventricular dysfunction. Our patient’s baseline kidney function was normal; however, the onset of AKI caused a rapid decline in renal clearance, exacerbating the cardiotoxic effects of flecainide. There are few published reports of flecainide toxicity secondary to renal impairment, but some case studies support this mechanism [6,8]. This highlights the importance of close monitoring of renal function in patients on flecainide therapy, as even transient reductions in kidney function can have significant clinical consequences.

Moreover, the link between flecainide and left ventricular (LV) systolic dysfunction is well-documented, primarily due to the drug’s sodium channel-blocking action affecting ventricular depolarization and myocardial conduction [3,4,5]. This interference can impair normal myocardial contractility, leading to reduced systolic function and eventual heart failure. Due to this risk, flecainide is contraindicated in patients with preexisting HFrEF or structural heart disease. The CAST trial (Cardiac Arrhythmia Suppression Trial) demonstrated increased mortality when flecainide was used in patients with structural heart disease or prior myocardial infarction [7]. Although large-scale studies quantifying the incidence of flecainide-induced HFrEF are lacking, reports suggest it is rare, particularly when used in patients without known cardiac disease. A retrospective study found that among over 400 patients treated with class Ic drugs, only a handful developed new systolic dysfunction, and even fewer had a direct temporal relationship with flecainide [9].

Interestingly, our patient had a normal ejection fraction and baseline kidney function before flecainide initiation and did not have underlying coronary artery disease or structural abnormalities. However, she developed HFrEF shortly after treatment initiation. The development of wide QRS with resultant septal dys-synchrony contributed to the reduction in ejection fraction. This reversible cardiomyopathy is believed to be induced by flecainide’s prolongation of conduction time, which impairs synchronized myocardial contraction [4,10]. The patient’s QRS duration improved from 192 ms to 130 ms after drug cessation, along with a concurrent improvement in EF, further supporting this mechanism. There have been isolated case reports of flecainide-induced cardiomyopathy and conduction disturbances like left bundle branch block in patients without preexisting cardiac disease, but they remain rare and underreported [4,8,11].

Therefore, even in patients with normal ejection fraction and no overt cardiac pathology, flecainide therapy should be approached with caution, particularly in older individuals or those with labile renal function. Some evidence suggests that elderly patients and females may have increased sensitivity to sodium channel blockers due to pharmacokinetic variability, although more data is needed [12,13]. Our case reinforces the need for individualized risk assessment, regular ECG monitoring, and kidney function surveillance during flecainide treatment, regardless of baseline risk stratification.

The decision to discontinue flecainide and initiate treatment with metoprolol succinate for rate control reflects the importance of promptly recognizing and addressing drug-induced adverse effects to optimize patient outcomes. This case also highlights the need for increased pharmacovigilance and research into pharmacogenomic factors that may predispose certain individuals to flecainide-related cardiac complications.

## Conclusions

This case report highlights the importance of considering flecainide-induced HFrEF as a potential adverse outcome in patients receiving antiarrhythmic therapy. Through a meticulous examination of the literature, we have explored the intricate mechanisms behind flecainide's action, its contraindications in HFrEF, and its potential to induce heart failure in patients with normal baseline cardiac and kidney function. Vigilance in patient monitoring and further research efforts, including the exploration of genetic factors and pharmacogenomics, are essential to refine risk stratification. This will enable a more nuanced approach to flecainide therapy, ensuring optimal cardiovascular outcomes for patients managing arrhythmias.
